# Analysis of Psychological Disorders and Adaptive Influence of Blended Learning of College Students

**DOI:** 10.1155/2022/5418738

**Published:** 2022-09-24

**Authors:** Yi Li

**Affiliations:** Graduate Institute of Henan University, Henan, Kaifeng 475004, China

## Abstract

College students' learning disabilities and learning adaptability refer to the fact that college students, on the basis of their own basic abilities and personality characteristics, actively make self-adjustments by interacting with their learning and learning environment problems in order to achieve a balance between the internal and external learning environment and the ability to be in a state of vigorous development. From the perspective of student development, the mastery of learning knowledge and skills directly affects the development of college students themselves, and the level of adaptability to learning directly affects or determines the effectiveness of college students' learning. From the perspective of social needs, society has higher requirements on the learning ability and learning skills of college students, and in talent selection, they tend to choose college students who study counterparts and have excellent grades. It can be seen that the learning of college students is of great significance and role to them, and the quality of college students' development in learning has a significant impact on their talent and growth. This study comprehensively proposes from multiple theoretical perspectives that five factors, including learning commitment, learning goals, learning behavior, learning self-efficacy, and learning external environment, will affect the level of college students' learning disabilities and learning adaptability. This study draws the following conclusions: (1) the influencing factors of college students' learning disabilities and learning adaptability are in descending order of average score: learning self-efficacy, learning commitment, learning behavior, learning goals, and learning external environment. The average scores of the factors of effectiveness and learning commitment are greater than the total average score of each factor, while the average scores of learning behavior, learning objectives, and special external environment factors are lower than the total average score of each factor. (2) The three factors of learning commitment, learning self-efficacy, and learning behavior can significantly affect the level of learning disability and learning adaptability, but learning goals and learning external environment have no significant effect on learning disability and learning adaptability.

## 1. Introduction

At present, human beings are in a new era with intelligence and informatization as the core features. In particular, with the successive emergence and wide application of new technologies such as cloud computing, big data, Internet of Things, mobile Internet, and artificial intelligence, the pace of informatization in various economic and social industries has been accelerating, and the overall informatization of society has continued to deepen. The revolutionary impact is becoming more and more obvious. These advanced technologies have transformed traditional teaching and learning methods, spawned new teaching methods, curriculum forms, and learning methods, and promoted further changes in educational informatization. Online open courses and blended learning have adapted to this change and have become a new form of education and teaching reform in today's colleges and universities. At present, the application of online open courses in colleges and universities mainly includes two forms of fully online learning and blended learning. Among them, blended learning, as a typical form of online open course application, has also become a new and important learning method in colleges and universities. It effectively breaks through the limitations of time and space and embodies the sharing of learning resources, the autonomy of learning methods, the integration of learning activities, the diversification of interactive behaviors, the integration of learning evaluation, and the informatization of learning management—the key force to promoting the reform of education and teaching in colleges and universities in today's era [1–6].

As the era of Internet technology and artificial intelligence gradually affects the field of higher education, MOOCs, known as “the greatest innovation in education since the invention of printing,” appeared. As a force for subversive change, MOOCs break the time and space boundaries of education and promote fundamental changes in traditional college classrooms. In this process of subversive innovation and change, traditional classrooms in colleges and universities are gradually being replaced with blended learning classrooms that combine online and offline, thus promoting the cultivation of innovative talents in colleges and universities. Blended learning has advantages over traditional classrooms. First of all, blended learning can better achieve teaching students in accordance with their aptitude. In traditional classrooms, there are many students, and students' learning mainly relies on the knowledge imparted by the teacher alone. Each student's learning ability and absorptive capacity are different, and their acceptance of teachers' teaching methods is also different, which directly affects students' learning efficiency. Through blended learning, teachers can use the online interaction with students to make up for the teaching problems that are not solved in offline classrooms, and students can also learn every knowledge point and increase the emotions of teachers and students. Second, blended learning is more flexible in teaching methods. Due to time and space constraints, traditional classrooms often have problems such as low teaching efficiency and low classroom participation. Through blended learning, teachers can solve the problem of low classroom participation through online platforms, using video, audio, and other forms. Finally, blended learning can make students happier while they learn. Traditional classrooms are often boring and dull, and students' interest in learning is easily affected. Through blended learning, teachers can use interactive teaching materials to interact with students, transform knowledge points into attractive videos, promote active learning and active thinking, and achieve the goal of easy and happy learning.

For every college student, learning is a multilevel and multifaceted adaptive psychological activity that enables the individual to gain experience and lead to adaptive changes in the individual's existing psychological structure. Adaptation is a process that almost every college student needs to go through. However, there are indeed some people who have various problems because they cannot adapt smoothly, which affects their normal studies and life. Especially in colleges and universities with a science and engineering background, there are some nonmainstream studies, and many students who should study do not have a high degree of recognition of learning. Some students even take the college entrance examination. There are serious problems in learning thoughts and emotions, showing lack of motivation for learning, lack of self-confidence, weariness, hesitation, being unable to adapt to university life well and feeling very confused, poor academic performance, or even dropping out of school, which bring great harm to the society and the family. The researchers feel that adaptability is an important ability of contemporary college students and maladaptation will have a negative impact on the individual's physical and mental aspects; on the contrary, good adaptability will have a positive and far-reaching impact on the individual's physical and mental aspects. The purpose of this research is to draw attention to college students' learning problems and continue to conduct in-depth research on college students' learning disabilities and learning adaptation. The third is to understand the students' learning adaptability and improve their learning adaptability, which is not only directly related to students' personal success and development but also related to their healthy psychological growth and the smooth completion of socialization, which has important social significance and far-reaching educational significance [7–14].

## 2. Related Work

The learning disability and learning adaptability of college students is a major field of adaptability research of foreign college students. In general, it mainly discusses two aspects: one is the main factors affecting the learning disability and learning adaptability of college students; the other is the testing tool for college students' learning disability and learning adaptability.

In terms of the main factors affecting college students' learning disabilities and learning adaptability, the researchers believe that there are two aspects: students' personality factors and environmental factors. In terms of personality factors, Chemers et al. explored the relationship between college students' academic self-efficacy, optimism and college students' learning disabilities and learning adaptability, psychological stress and health, and academic achievement. The study found that academic self-efficacy, optimism tendency, and coping ability were significantly positively correlated with college students' academic expectations, learning disabilities, and learning adaptability. Rice et al. studied the relationship between college students' self-esteem and learning disabilities and learning adaptability. The results show that the self-esteem level of college students has a significant positive correlation with learning disabilities and learning adaptability. Chartran, Judy studied the impact of college students' personality factors on their learning disabilities and learning adaptability from the perspectives of social psychology and human-environment adaptation. The research shows that college students' self-evaluation, and sense of responsibility for learning and becoming an excellent student are expected from college students, which will directly affect the learning disability and learning adaptability of college students in the first academic year and further affect their later learning adaptability and academic achievement. As far as environmental factors are concerned, a large number of studies have focused on the impact of college students' family factors on their learning disabilities, learning adaptability, and academic achievement. These studies show that parents are still an important factor affecting students' socialization, and the relationship between parents and students is an important indicator for predicting students' adjustment to college life. In addition, the researchers also explored the problems of learning disabilities and learning adaptability of college students from the aspects of gender, difficulty of learning tasks, school discipline, teaching interaction, dormitory environment, library materials, and Internet information utilization [15–20].

In addition to analyzing the influencing factors, foreign scholars also discussed the influence of other factors on learning disabilities and learning adaptability. Research has shown that older students have more traits that help them succeed, such as high levels of motivation, persistence, and responsibility, as well as adaptability to different learning environments and an independent approach to learning. Some scholars have found that students with high persistence are different from those with low persistence in self-evaluation, which affects students' learning adaptation and academic performance differences. Rebecca et al. explored the relationship between individual adaptability and personality and conducted a longitudinal study of up to 20 years. They followed up on 205 children and evaluated them when they were 10, 20, and 30 years old. It was found that children's different personalities predicted their adaptive status and changes in these three age groups. Conversely, characteristics of adult personality can also be predicted from childhood adaptations, and in some cases, child adaptations predict adult personality development. Personality traits such as high levels of motivation and self-awareness in learning during childhood will affect children's academic performance, attitude, and future work performance. A passionate and agreeable personality is closely related to adaptability. At the same time, researchers have also carried out some research on the assessment tools of college students' learning disabilities and learning adaptability. Among them, most of the assessments of college students' learning disabilities and learning adaptability are included in the overall adaptability of college students. The College Student Adaptability Level Questionnaire (CARS) developed by Zitow is used to study the subjective assessment of college students when they face stressful events in college life, including the assessment of learning stressful events. Simon et al. compiled the College Students' Response and Adaptation Questionnaire (TRAC) for college freshmen's learning adaptation, which divided learning disabilities and learning adaptability into belief (Belief), emotion (Emotional), and behavioral (Behavioral) as the basic dimensions. The nine factors in the study are fear of failure, test anxiety, test preparation, attention to quality, peer help, teacher help, learning priorities, effective learning methods, and ease of learning.

In general, because the problem of learning adaptation is most prominent at the beginning of admission, in foreign research, most researchers limit learning adaptation to the beginning of admission and do not discuss the learning adaptation of middle and senior college students. It mainly discusses self-efficacy, learning attitude, self-evaluation, and sense of responsibility and even includes the individual characteristics of students and the relationship between parents and students. Foreign research on influencing factors is more scattered and special. At the same time, foreign studies on learning disabilities and learning adaptability are mainly aimed at some special student groups, such as immigrant students, refugee children, and gifted students. The research is often more sociological and anthropological in nature.

## 3. Construction of a Model Based on Mixed Learning Mental Disorders and Adaptability of College Students

### 3.1. Definition of Research Variables

#### 3.1.1. Definition of Learning Disabilities and Adaptability of College Students

According to the above concepts, such as adaptation, adaptability, college student adaptability, college student learning disability, and learning adaptability, we further discuss the concepts of college student learning disability and learning adaptability. The research scope of college students' learning disability and learning adaptability is wider, involving learning, extracurricular knowledge learning, and other skills learning. It is the learning adaptability under the influence of the whole school learning atmosphere, and it is a kind of learning adaptability formed under the influence of specific school spirit and school discipline. The unique learning adaptability reflects a macro idea; compared with the learning disabilities and learning adaptability of college students, the learning disabilities and learning adaptability of college students are more microscopic, reflecting the adaptability of a learning student and refining the learning of the learning. Education, students' adaptation, and the learning adaptability of different learning are affected by the environment, such as learning atmosphere and learning cultural atmosphere [21]. College students' learning disabilities and learning adaptability refer to the fact that college students, on the basis of their basic abilities and personality characteristics, actively make self-adjustments by interacting with the learning and learning environment in order to achieve a strong development of a balanced internal and external learning environment. This definition emphasizes that in the process of learning adaptation, the individual's self-cognition, behavior, and psychological adjustment are mainly affected by subjective behavior self-adjustment and learning external environment. The subjective behavior self-adjustment includes learning commitment, learning goals, Learning behavior, learning coping, and learning self-efficacy; learning external environment includes student comparison, subject environment, and employment environment.

#### 3.1.2. The Framework of Self-Adjustment of Subjective Behavior

This study believes that college students' learning disability and learning adaptability are a kind of psychological and ability tendencies formed by college students in the process of learning. They are mainly affected by the self-adjustment of subjective behavior and the adaptation state of the learning environment. The self-adjustment of subjective behavior includes learning commitment, learning goals, learning behaviors, learning to cope, and learning self-efficacy, each of which may contain 2–4 factors: learning commitment, starting from students' metacognition of learning, measuring students' beliefs, cognitions, emotional attachment, willingness to devote time and energy to learning, and self-awareness of desire to be successful in learning. It includes three factors: learning identification commitment (learning interest), learning commitment, and learning development commitment. Learning goals, starting from intrinsic needs, measure the goal-oriented intensity and autonomy of students' choice and learning, including learning motivation and learning goals (2 factors).

Learning behavior, starting from the students' actual learning behavior and performance, measures the students' performance in learning behavior, including four factors: the use of learning methods, the use of learning resources, the use of knowledge, and the preparation for career selection. Learning coping measures the coping behaviors and strategies of college students when they experience stress and stress in the learning process. It includes three factors: cognitive coping, emotional coping, and behavioral coping. Learning self-efficacy is the evaluation of students' own learning ability and behavior at the cognitive level, including the evaluation of their own learning competence and learning behavior, as shown in Table 1.

#### 3.1.3. Learning the Architecture of the External Environment

The external environment of learning includes student comparison, subject environment, and employment environment. Student comparison, in the same university, starts from the mutual attitudes of different students to measure the psychological reflection of the students in the case of other students' bad attitudes. Especially in colleges and universities, the attitudes and opinions of mainstream students toward nonmainstream students may have a greater impact on the learning disabilities and learning adaptability of nonmainstream students. Subject environment, within the same university, from the perspective of the subject status of the study in the whole school, the psychological reflection of students due to the subject status of learning, and the subject status of learning will also directly affect the students' learning disabilities and learning adaptability. Employment environment: within the same university, nonmainstream learning also has certain disadvantages in terms of employment. From the perspective of employment after graduation, the psychological reflection of students in the face of employment difficulties is measured. The specific meaning of each factor is as follows (see Table 2) [22].

### 3.2. Research Hypothesis

The study of college students' learning disabilities and learning adaptability is further discussed based on the related researches on adaptation, adaptability, college students' adaptability, college students' learning disabilities, and learning adaptability. Through literature review, most scholars discuss the influencing factors of learning disability and learning adaptability from the aspects of learning motivation, learning method, teaching mode, learning attitude, learning environment, physical and mental adaptation, self-efficacy, and so on. There are also studies on learning commitment, learning burnout, learning style, self-efficacy, and other aspects of college students' learning disabilities and learning adaptability:

1. The learning commitment intensity of college students reflects the learning disabilities and learning adaptability of college students to a certain extent. Improving college students' learning identity is an important issue to promote college students to form positive learning psychology and reduce their negative psychology. The degree can affect the learning disability and learning adaptability of college students.
2. In China, it is generally believed that college students' learning burnout is a negative attitude and behavior in that they feel tired of learning due to learning pressure or lack of learning goals and interests. This study believes that the most fundamental reason for the phenomenon of college students' learning burnout is their incompatibility with learning. The reasons for this result are learning goals and learning behaviors. If students have strong learning interests and positive learning behaviors, they will have good adaptability; therefore, learning goals and learning behaviors positively affect learning disabilities and learning adaptation to a certain extent.
3. Chemers et al. explored the relationship between college students' academic self-efficacy and college students' learning disabilities and learning adaptability. The study found that academic self-efficacy and learning disabilities were significantly positively correlated with the level of learning adaptability. We believe that self-efficacy can positively affect learning disabilities and learning adaptability to a certain extent.
4. Bornefnbremler (Bornefnbremler) proposed from the ecological theory of human development that a person's growth process will constantly adapt to the changing surrounding environment in which to live. This process is affected by the direct and indirect influence between people and the surrounding environment and the environment, as well as the social background on which the surrounding environment relies; that is to say, the environment will also have an impact on people's behavior. Therefore, we believe that the external environment will affect learning disabilities and learning adaptability.

**Figure 1:**
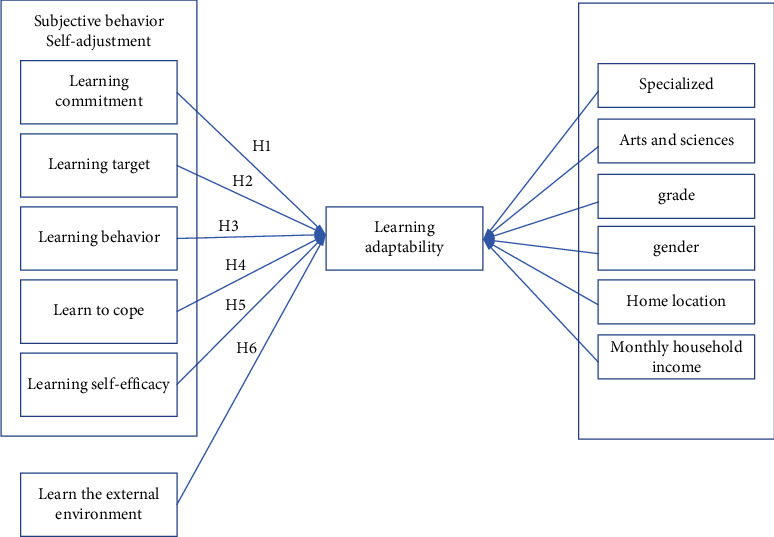
Research model of learning disabilities and learning adaptability.

Suppose the following:

① College students' learning disabilities and learning adaptability are psychological and behavioral tendencies.
② College students' learning disabilities and learning adaptability are a multilevel structure. The direct influencing factors of college students' learning disabilities and learning adaptability are mainly composed of subjective behavior self-adjustment and learning external environment. Among them, subjective behavior self-adjustment includes learning commitment, learning goals, learning behavior, learning coping, and learning self-efficacy; each subscale is divided into 3-4 factors; the external learning environment includes student comparison, academic environment, and employment environment.
③ The learning disabilities and learning adaptability of college students have certain developmental rules, and there are differences in gender, learning type, liberal arts and sciences, source of students, and grades.
④ Both subjective behavior self-adjustment and learning environment adaptation state affect learning disability and learning adaptability. Among them, self-adjustment of subjective behavior positively affects learning disability and learning adaptability, and the external environment of learning also positively affects learning disability and learning adaptability, as shown in Figure 1.
    H1: Learning commitment is positively related to learning disability and learning adaptability
    H2: Learning goals are positively related to learning disabilities and learning adaptability
    H3: Learning behavior is positively related to learning disability and learning adaptability
    H4: Learning coping is positively related to learning disability and learning adaptability
    H5: Learning self-efficacy is positively correlated with learning disability and learning adaptability
    H6: Learning the external environment is positively related to learning disability and learning adaptability

## 4. Analysis of Learning Psychological Barriers and Adaptive Factors Based on Blended College Students

### 4.1. Data Collection

In this study, 280 questionnaires were distributed in the form of paper questionnaires, and a total of 251 questionnaires were recovered, of which 245 were valid questionnaires, and the effective recovery rate was 87.5%. In this study, the researchers mainly used the following methods to eliminate invalid questionnaires: (1) eliminate questionnaires with more than 95% of the items being the same answer.

The six main factors obtained by the principal component analysis of the collected data are one less variable than the influencing factors proposed by the earliest research model. There is a good fit with the observed data. The learning coping factor is classified into the learning behavior factor, which is more in line with the actual situation of college students' learning adaptation. The questions on the learning commitment factor include T01, T04, T07, and T08; the questions on the learning goal factor include T03, T09, T21, and T36; the questions on the learning behavior factor include T05, T23, and T27; the questions on the learning disability and learning adaptability factors include T13, T15, T18, T24, T25, and T29; the questions on the learning self-efficacy factors include T31, T32, T35, and T37; the questions on the learning external environmental factors include T39, T40, and T41.

**Figure 2:**
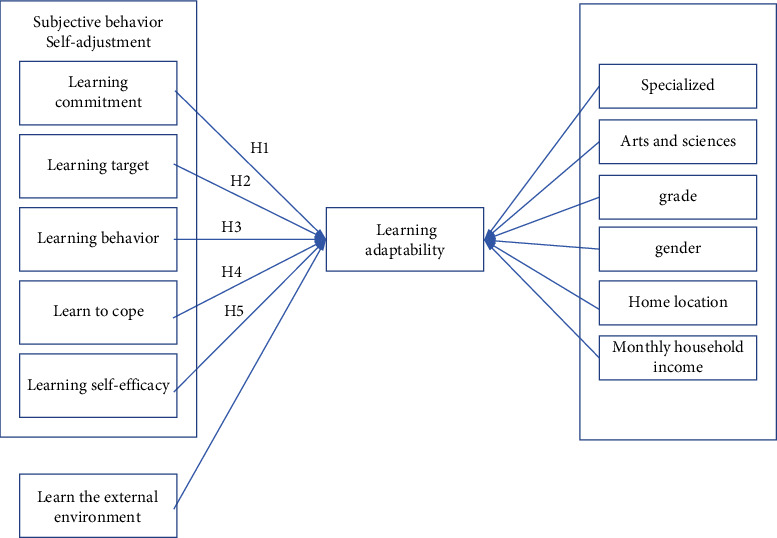
Revised research model.

According to the above situation obtained by factor analysis, “learning coping” is included in “learning behavior” as one of its subvariables. This study makes amendments to the research model proposed in 3.2. The revised research model is shown in Figure 2.

Therefore, the research hypothesis is also revised as follows:

  H1: Learning commitment is positively related to learning disability and learning adaptability
  H2: Learning goals are positively related to learning disabilities and learning adaptability
  H3: Learning behavior is positively related to learning disability and learning adaptability
  H4: Learning self-efficacy is positively correlated with learning disability and learning adaptability
  H5: Learning the external environment is positively related to learning disability and learning adaptability

### 4.2. Correlation Analysis of Research Models

Correlation analysis can effectively reveal the strength of the statistical relationship between the research variables. The correlation of research variables means that there is a certain relationship between the two in the direction and magnitude of development and change. The correlation coefficient *r* is the most widely used statistic to measure the strength of association between research variables, and its value ranges from 0 to ±1, with 0 indicating no correlation and ±1 indicating complete correlation. This study will conduct correlation analysis on the corresponding variables according to the relationship of each group of variables proposed by the research model.

According to the hypothesis of the research model, there are three groups of influencing factors for “learning disability and learning adaptation,” namely “subjective behavior self-adjustment,” “learning external environment,” and “student personal factors.” Sex is the dependent variable, “subjective behavior self-adjustment” and “learning external environment” are the independent variables, and “student personal factors” are the control variables. Therefore, this study firstly conducted a correlation analysis between the above independent variables and dependent variables.

It can be seen from Table 3 that, considering the mutual influence of each factor, if the significance level *α* of the two influencing factor variables is less than 0.05, it can be considered that there is a linear relationship, and both are positively correlated.

The correlation coefficient between learning disability, learning adaptability, and learning self-efficacy is 0.403, and the significance level *α* is less than 0.01. The correlation coefficient between learning disability and learning adaptability and learning commitment is 0.435, and the significance level *α* is less than 0.01. The correlation coefficient between adaptability and learning external environment is 0.127, and the significance level *α* is less than 0.05. The correlation coefficient between learning disability and learning adaptability and learning goals is 0.214, and the significance level *α* is less than 0.01. The correlation coefficient between learning disability and learning adaptability and learning behavior is 0.218, and the significance level *α* is less than 0.01. It can be seen that learning disabilities and learning adaptability are significantly correlated with the other five independent variables, and the correlation degrees are learning commitment (0.435) > learning self-efficacy (0.403) > learning behavior (0.218) > learning goals (0.214) > learning the external environment (0.127). The correlation coefficient between learning self-efficacy and learning commitment is 0.443, and the significance level *α* is less than 0.01. The correlation coefficient between learning self-efficacy and learning external environment is 0.074, and the significance level *α* is greater than 0.05. The correlation coefficient between learning self-efficacy and learning goals is 0.279, and the significance level *α* is less than 0.01. The correlation coefficient between learning self-efficacy and learning behavior is 0.177, and the significance level *α* is less than 0.01. The order of correlation is as follows: learning commitment (0.443) > learning disability and learning adaptability (0.403) > learning behavior (0.177) > learning goal (0.279). There is no significant correlation between learning self-efficacy and learning external environment.

Student self-efficacy is the internal driving force for students to generate autonomous learning motivation. To a large extent, students' learning motivation plays an important role in activating and strengthening students' learning behaviors and ensuring the effectiveness of students' learning behaviors and activities. Students with a strong sense of efficacy usually have a positive view of learning and also believe that they have strong learning ability, so they often have a strong sense of self-competence and believe that their educational ability can be continuously improved in learning development and thus often produces a facilitative, adaptive motivation to learn. Such students tend to set and choose challenging tasks and goals for themselves and actively strive to orient their behaviors and activities toward these goals. If you work hard and stick to it, it will also promote the improvement of students' learning behavior. Students with a weak sense of efficacy often doubt their own abilities, lack self-confidence in their learning ability and influence, and do not believe that this ability can be changed and improved through hard work, so they are often more likely to have obstructive learning motivations, are reluctant to choose aggressive goals, lack work initiative and enthusiasm, lack confidence and powerlessness in the face of difficulties, and even often exaggerate or avoid difficulties. [23] At the same time, due to the low self-belief and self-expectation of one's own educational ability, it often leads to low satisfaction, and it is also easier to reduce one's own learning commitment, showing frequent absenteeism and becoming a source of resistance to personal or organizational progress. The correlation coefficient between learning commitment and learning external environment is 0.130, and the significance level *α* is less than 0.05. The correlation coefficient between learning commitment and learning goals is 0.263, and the significance level *α* is less than 0.01. The correlation coefficient between learning commitment and learning behavior is 0.151, and the significance level *α* is less than 0.05. the order of correlation is as follows: learning self-efficacy (0.443) > learning disability with learning adaptability (0.435) > learning goals (0.263) > learning behavior (0.151) > learning external environment (0.130). The correlation coefficient between learning external environment and learning goals is 0.047, and the significance level *α* is greater than 0.05, and this correlation is not significant. The correlation coefficient between learning external environment and learning behavior is 0.077, the significance level *α* is greater than 0.05, and the correlation is not significan. The order of the degree of correlation is as follows: learning commitment (0.130) > learning disability and learning adaptability (0.127). There is no significant correlation between learning external environment and learning behavior, learning goals, and learning self-efficacy.

Students often feel a sense of loss, which is often manifested in weak beliefs, cognition, and emotional attachment to learning. Students are unwilling to invest time and energy in learning, and they are not eager to succeed in learning. This environment undoubtedly affects students' learning and adversely affects adaptation and other psychological adaptations.

The correlation coefficient between learning goals and learning behavior is 0.375, and the significance level *α* is less than 0.01. The order of correlation is as follows: learning behavior (0.375) > learning self-efficacy (0.279) > learning commitment (0.263) > learning disability and learning adaptability (0.214). There is no significant correlation between learning goals and learning external environment.

The degree of correlation between learning behavior and other factors is as follows: learning goals (0.375) > learning disabilities and learning adaptability (0.218) > learning self-efficacy (0.177) > learning commitment (0.151). There is no significant correlation between learning behavior and learning the external environment.

It can be seen that the dependent variable learning disability and learning adaptability are significantly correlated with the independent variables “subjective behavior self-adjustment” and “learning external environment,” and “subjective behavior self-adjustment” is more significant than “learning external environment.” “Subjective behavior self-adjustment” has significant pairwise correlations between the four factors, which fully demonstrates the relevance of subjective behaviors. “Learning external environment” is only significantly correlated with learning disabilities, learning adaptability, and learning commitment, and the degree of significance is not high, indicating that when considering the independent variable “subjective behavior self-adjustment,” the external learning environment will affect students' learning disability and learning adaptability and will also positively affect learning commitment.

### 4.3. Research Hypothesis Testing: Regression Analysis

Through the above correlation analysis, we can clarify the closeness and direction of the relationship between the variables, but this does not mean that there must be a causal relationship between the variables. Regression analysis can further clarify the direction of the variable relationship and verify whether there is a causal relationship between variables. Regression analysis, as a quantitative analysis method, is mainly used to analyze the statistical relationship between things, focusing on the quantitative change law between variables and describing and reflecting this relationship in the form of regression equations so as to accurately grasp that variables are affected by other variables or the degree of influence of multiple variables. In this section, regression analysis will be used to test the relevant hypotheses proposed by the research model. Whether the research data are suitable for regression analysis needs to be determined by the residual, collinearity, and linear relationship between the data. The established regression model is suitable and effective only if the research data conforms to the above-mentioned assumptions. In this study, path analysis will be performed through multiple regression, the linear relationship between the data will be tested through the scatter plot of residuals, and whether the data obeys the normal distribution will be tested through the histogram of residuals and cumulative probability plot, as shown in Table 4.

As can be seen from Table 5, when the five variables predict the learning disability and learning adaptability of college students, three variables enter the regression equation, which are learning self-efficacy, learning commitment, and learning behavior. The independent variable learning self-efficacy is significant, and the standardized regression coefficient is 0.231, which can effectively explain the dependent variable learning disability and learning adaptability; the independent variable learning commitment is significant, and the standardized regression coefficient is 0.317, which can effectively explain the dependent variable learning disability and learning adaptability; the independent variable learning external environment is not significant, and the standardized regression coefficient is 0.075; the independent variable learning target is not significant, and the standardized regression coefficient is −0.020; the independent variable learning behavior is significant and can be used to predict the factor variables (learning disability and learning adaptability), and the standardized regression coefficient was 0.144. The three independent variables of learning commitment, learning self-efficacy, and learning behavior can be significantly used to predict the dependent variable (learning disability and learning adaptability); however, on average, learning goals and learning external environment cannot predict the dependent variables (learning disability and learning adaptation).

**Figure 3:**
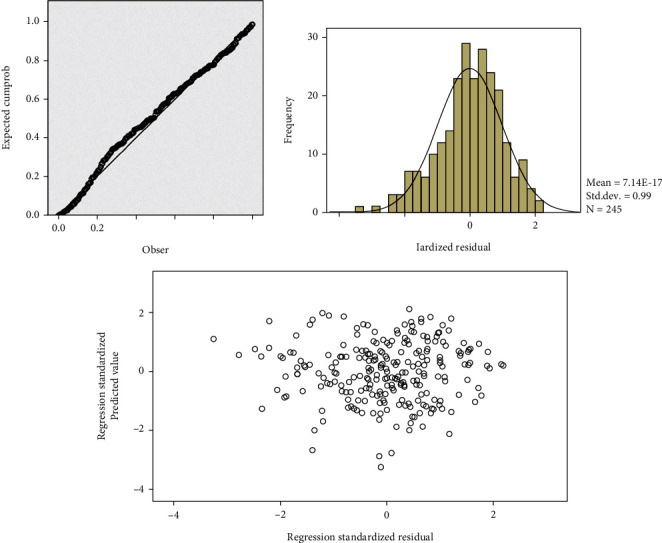
Residual scatter plot, histogram, and cumulative probability plot of learning disability and learning adaptability.

Research shows that college students have a certain sense of identity and emotional attachment to what they have learned and are willing to invest in their learning. Investing a certain amount of time and energy in learning is the most effective way to improve learning disabilities and learning adaptability. At the same time, whether students can judge the degree of matching between their own personality traits and learning and whether they can adopt certain learning methods to achieve learning goals can also effectively predict students' learning disabilities and learning adaptability. But the findings also tell us that, on average, learning the external environment does not predict learning disabilities and learning adaptations [24]. The reason may firstly be that this questionnaire was designed by researchers on the basis of different scales at home and abroad, and there are many deficiencies that need further research; secondly, the relationship between the two variables obtained through regression equation analysis is not considered. Other variables affect them that learning disability and learning adaptability, which are a kind of psychological aptitude, and students' learning adaptability level is more affected by internal factors. More high-achieving students are active in the class, and they excel more through their own efforts. Therefore, when the external environment of learning and the relationship between the two variables of learning disability and learning adaptability are simply considered, it is also possible that the external environment cannot predict learning disability and learning adaptability, as shown in Figure 3.

### 4.4. Arrangement of Research Hypotheses and Path Analysis of Research Models

Based on the literature review and theoretical analysis, we first proposed 6 sets of hypotheses based on the research model. After the reliability and validity analysis, we revised the research model according to the results and adjusted the research hypothesis to 5 groups. Finally, we verified each group of hypotheses through linear regression analysis and organized the results into the following Table 6.

**Figure 4:**
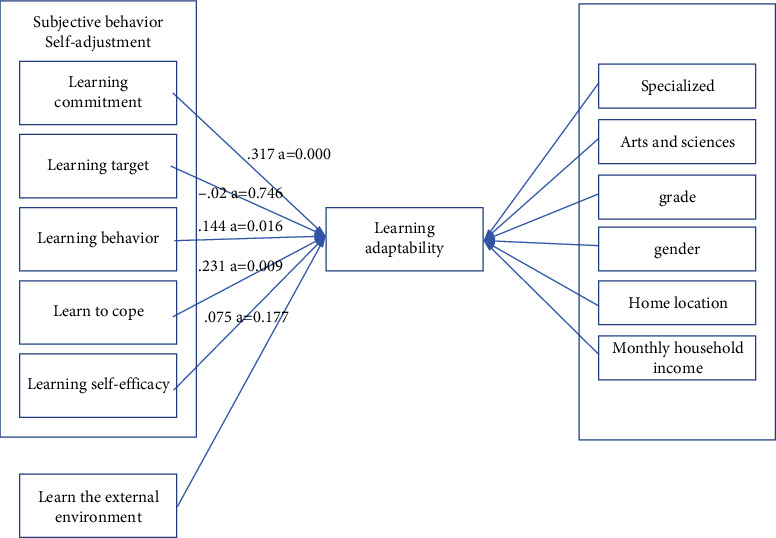
Research model roadmap.

We then complete the path analysis of the research model based on the analysis data above. The path coefficient of the path diagram adopts the standardized regression coefficient *β* in the regression analysis, as shown in Figure 4.

Equation: *f* (learning disability and learning adaptability) = 0.317 ×  (learning commitment) + (−0.020) × (learning goal) + 0.144 × (learning behavior) + 0.231 × (learning self-efficacy) + 0.075 × (learning external environment).

## 5. Conclusion

This exploratory study on college students' learning disabilities and learning adaptability constructed a research model that affects college students' learning disabilities and learning adaptability, compiled a college student's learning disability and learning adaptability assessment scale, and carried out specific research on college students' learning adaptability problems. The study found the following results.

The overall characteristic scores of college students' learning disability and learning adaptability and their influencing factors showed a trend. The order of the average value of each factor was as follows: learning self-efficacy (3.01) > learning commitment (2.99) > learning disability and learning adaptability (2.83) > Learning behavior (2.53) > learning objective (2.42) > learning external environment (2.38), in which the average score of learning self-efficacy, learning commitment, learning disability, and learning adaptability factor is greater than the average item score (2.73) of learning behavior and learning goal. The average score of the dedicated external environment factor is less than the average score of the project. This shows that college students have a certain sense of identity and emotional attachment to what they have learned, are willing to invest a certain amount of time and energy in learning, have a certain intensity of internal motivation and interest in learning, and can make a certain judgment on the degree of matching of learning, but they are affected by external learning. The negative influence of the environment, the learning motivation may not keep up with the emotional commitment to learning and the internal need for learning, the learning goals are not clear, and the unsatisfactory performance in learning behavior is out of sync with the learning commitment. Motivation and learning the external environment adaptation need to be guided and promoted; especially, the adaptation state of learning the external environment needs to be solved urgently.

## Figures and Tables

**Table 1:** The meaning of each factor of subjective behavior self-adjustment.

Dimension	Secondary factor	Variable meaning
Learning commitment	Learning identity (learning interest)	Students have a correct and comprehensive understanding of learning, are enthusiastic and interested in what they have learned, emotionally identify and accept what they have learned, and are emotionally attached to learning
	Learning input	Willingness to invest time, energy, etc.
	Learning development	The student's willingness to continue learning and a desire to learn to develop a desire to learn

Learning goals	Motivation to learn	Behavioral reasons and motivational strength for individuals to achieve good academic performance
	Learning target	Have clear and reasonable goals for learning and constantly adjust learning goals

Learning behavior	Method usage	According to the learning requirements and own characteristics, formulate a reasonable plan, form a learning method suitable for oneself, and carry out effective learning
	Resource utilization	Effective use of learning resources such as library, Internet, experimental equipment, and teacher resources
	Knowledge application	Apply the learning theory and knowledge learned to real life, linking theory with practice

Learn to cope	Cognitive coping	When learning to adapt to difficulties, evaluate and recognize the problem and reconstruct the cognitive
	Emotional correspondence	Learn to mediate and control the emotions or behaviors that arise when they encounter difficulties
	Behavioral coping	When encountering difficulties in the learning and adaptation process, take action to effectively cope with the problem

Learning self-efficacy	Learning trait efficacy	Students' judgment and confidence in their professional values, interests, abilities, temperament, and the degree to which they match their studies
	Learning effectiveness	Students' judgment and confidence in whether they can use certain learning methods to achieve their learning goals

**Table 2:** The meaning of each factor in the learning external environment.

Dimension	Secondary factor	Meaning of variables
Learn the external environment	Student comparison	Under the influence of other students' bad attitude, students' psychological reflection
	Disciplinary environment	When the subject status of the study is not strong in the whole school, the students' psychological reflection
	Employment environment	Under the circumstance that the advantages of learning and employment are not obvious, the psychological reflection of students

**Table 3:** Correlation analysis table between factors.

		Learning adaptability	Learning self-efficacy	Learning commitment	Learning the external environment	Learning target	Learningbehavior
Learning adaptability	Related analysis	1.000					
	Salience	.					
	Number of scales	245					

Learning self-efficacy	Related analysis	0.403^*∗∗*^	1.000				
	Salience	0					
	Number of scales	245	245				

Learning commitment	Related analysis	0.435^*∗∗*^	0.443^*∗∗*^	1.000			
	Salience	0	0	.			
	Number of scales	245	245	245			

Learn the external environment	Related analysis	0.127^*∗*^	0.074	0.130^*∗*^	1.000		
	Salience	0.046	0.250	0.042			
	Number of scales	245	245	245	245		

Learning target	Related analysis	0.214^*∗∗*^	0.279^*∗∗*^	0.263^*∗∗*^	0.047	1.000	
	Salience	0.001	0	0	0.461		
	Number of scales	245	245	245	245	245	

Learning behavior	Related analysis	0.218^*∗∗*^	0.177^*∗∗*^	0.151^*∗∗*^	0.077	0.375^*∗∗*^	1.000
	Salience	0.001	0.006	0.018	0.231	0.000	
	Number of scales	245	245	245	245	245	245

^*∗∗*^Significant correlation at the 0.01 level. ^*∗*^Significant correlation at the 0.05 level.

**Table 4:** ANOVA table for the applicability of regression analysis.

Model		Sum of squares	Degrees of freedom	Mean square		Salience
1	Regression equation	66.831	5	13.366	18.031	0.000 (a)
	Residual	177.169	239	0.741		
	Sum	244	244			

^a^Independent variables: learning behavior, learning external environment, learning self-efficacy, learning goals, and learning commitment. ^b^Dependent variable: learning disability and learning adaptability. As can be seen from the above table, the significance level of the regression equation is <0.01, so it is meaningful to use 5 independent variables to predict 1 dependent variable.

**Table 5:** Regression analysis coefficient table.

Model		Unstandardized regression coefficients		Standardized regression coefficients		Salience
		*B*	Standard error	Beta	*B*	Standard error
1	Constant	1.92*E* − 16	0.055		0.000	1
	Learning self-efficacy	0.231	0.063	0.231	3.644	0.000
	Learning commitment	0.317	0.064	0.317	4.975	0.000
	Learning the external environment	0.075	0.055	0.075	1.355	0.177
	Learning target	−0.020	0.062	−0.020	−0.325	0.746
	Learning behavior	0.144	0.060	0.144	2.419	0.016

**Table 6:** Hypothesis testing results of the research model.

Hypothetical content	Test result
H1: Learning commitment will significantly and positively affect students' learning adaptability	Accept
H2: Learning goals will significantly and positively affect students' learning adaptability	Do not accept
H3: learning behavior will significantly and positively affect students' learning adaptability	Accept
H4: Learning self-efficacy will significantly and positively affect students' learning adaptability	Accept
H5: Learning the external environment will significantly and positively affect students' learning adaptability	Do not accept

## Data Availability

The dataset can be accessed upon request to the corresponding author.
